# When very low event rates limit the interpretation of non-inferiority trials

**DOI:** 10.1016/j.lansea.2026.100868

**Published:** 2026-09-09

**Authors:** Nitin Gupta, Praveen K. Tirlangi, Martin P. Grobusch

**Affiliations:** a Department of Infectious Diseases, Kasturba Medical College, Manipal Academy of Higher Education, Manipal, Karnataka, India; b Centre of Tropical Medicine and Travel Medicine, Department of Infectious Diseases, Amsterdam University Medical Centres, Location AMC, University of Amsterdam, Amsterdam, Netherlands

Baharia and colleagues compared 7-day and 14-day low-dose primaquine for radical cure of *Plasmodium vivax* malaria in India.^1^ The study addresses an important operational question, but the very low number of recurrences warrants caution when interpreting the non-inferiority conclusion.

The trial assumed a recurrence incidence of about 13%, an absolute non-inferiority margin of 10 percentage points, and 68% power. In practice, only four recurrences occurred among 400 participants over 180 days, two in each group, giving a cumulative incidence of 1.1% in both arms and a hazard ratio of 0.96 (95% CI 0.13–6.80).

When observed risks are only about 1%, an absolute margin of 10 percentage points can be satisfied despite very limited outcome information. The wide hazard-ratio confidence interval is consistent with substantial benefit or a markedly higher recurrence hazard, illustrating the imprecision resulting from only four events. In time-to-event analyses, information is driven primarily by the number of events rather than enrolment.^2^

This does not imply that the prespecified analysis was incorrect, nor that the authors overlooked this limitation. Rather, meeting a non-inferiority margin is not equivalent to providing precise evidence of comparable efficacy.^3^ The findings reassuringly show similarly low recurrence over 180 days, but provide limited ability to exclude a clinically important difference between regimens. This distinction is particularly relevant because the realised event rate was far below that assumed in the design. Longer follow-up and trials accruing sufficient recurrence events would strengthen the evidence, particularly where late relapses are expected.

## Contributors

NG conceived and drafted the letter. PKT and MPG critically revised it. All authors approved the final version and are accountable for the work.

## Declaration of interests

We declare no competing interests.
